# Fabrication and photoactivity of ionic liquid–TiO_2_ structures for efficient visible-light-induced photocatalytic decomposition of organic pollutants in aqueous phase

**DOI:** 10.3762/bjnano.9.54

**Published:** 2018-02-14

**Authors:** Anna Gołąbiewska, Marta Paszkiewicz-Gawron, Aleksandra Sadzińska, Wojciech Lisowski, Ewelina Grabowska, Adriana Zaleska-Medynska, Justyna Łuczak

**Affiliations:** 1 University of Gdansk, Faculty of Chemistry, Department of Environmental Technology, Wita Stwosza 63, 80-308 Gdansk, Poland; 2 Gdansk University of Technology, Chemical Faculty, Department of Chemical Technology, Narutowicza 11/12, 80-233 Gdansk, Poland,; 3 Institute of Physical Chemistry, Polish Academy of Sciences, 01-224 Warsaw, Poland

**Keywords:** heterogeneous photocatalysis, ionic liquids, TiO_2_, visible light catalysis

## Abstract

To investigate the effect of the ionic liquid (IL) chain length on the surface properties and photoactivity of TiO_2_, a series of TiO_2_ microspheres have been synthesized via a solvothermal method assisted by 1-methyl-3-octadecylimidazolium chloride ([ODMIM][Cl]) and 1-methyl-3-tetradecylimidazolium chloride ([TDMIM][Cl]). All as-prepared samples were characterized by X-ray powder diffraction (XRD), X-ray photoelectron spectroscopy (XPS), diffuse reflectance spectroscopy (DRS), scanning transmission microscopy (STEM) and the Brunauer–Emmett–Teller (BET) surface area method, whereas the photocatalytic activity was evaluated by the degradation of phenol in aqueous solution under visible light irradiation (λ > 420 nm). The highest photoefficiency (four times higher than pristine TiO_2_) was observed for the TiO_2_ sample obtained in the presence of [TDMIM][Cl] for a IL to TiO_2_ precursor molar ratio of 1:3. It was revealed that interactions between the ions of the ionic liquid and the surface of the growing titanium dioxide spheres results in a red-shift of absorption edge for the IL–TiO_2_ semiconductors. In this regard, the direct increase of the photoactivity of IL–TiO_2_ in comparison to pristine TiO_2_ was observed. The active species trapping experiments indicated that O_2_^•−^ is the main active species, created at the surface of the IL–TiO_2_ material under visible-light illumination, and is responsible for the effective phenol degradation.

## Introduction

The development of heterogeneous photocatalysis to degrade organic pollutants in aqueous and gas phases requires visible-light responsive, stable materials and a basic understanding of these materials [1–4]. Although various semiconductors are considered for environmental pollution abatement, titanium dioxide (TiO_2_) is still the most promising due to its stability, low cost, nontoxicity and availability [5]. Despite many advantages, the commercial application of TiO_2_ to solve environmental problems is still limited. The main obstacle is the relatively wide band gap of 3.2 eV for anatase that limits the photoexcitation wavelength needed to activate photocatalytic reactions to ultraviolet (UV) irradiation [6]. To break through the aforementioned drawbacks, semiconductor coupling [7], sensitization by inorganic complexes or organic dyes [8–9], as well as metal nanoparticle (NP) deposition [10] and metal doping [11] have been applied and have revealed potential to achieve visible-light-activated photocatalysts.

Another, actually surprising, way to improve the efficiency of solar-driven photocatalysis appeared in an application of ionic liquids (ILs) for TiO_2_ preparation [12–14]. Sometimes referred to as “solvents of the future”, ILs have induced a large and still growing interest from the scientific community and continuously find new areas of application. This scientific interest comes from the many interesting properties such as negligible vapor pressure, high thermal and electrochemical stability, and inflammability, providing a neoteric media for nano- and microstructure preparation with novel properties [15]. Especially, their polarity and affinity towards particles and precursors, transport and surface properties seem to be crucial for formation of the protective layer at the particle surface, thus electrosteric, solvation and viscous stabilization of growing particles [15–18].

The initial works on the application of ILs for semiconductor (including TiO_2_) preparation were focused mainly on the usage of these salts as solvents (reaction medium) [19–21] or components of the reaction system [22–25]. Their role was devoted to control the crystallization process and formation of the final particle structure (structuring agent). In this regard, various interesting structures were obtained and their properties are described in the literature. However, the more advanced works presented examples of the practical usage of the IL–TiO_2_ composites, for example, for photo-electrochemical water splitting [26–27], photo-oxidation of benzyl alcohol [12], phenol [28], degradation of chlorophenol [17,29], reduction of Cr^6+^ to Cr^3+^ [30] and the photocatalytic desulfurization of diesel oil [31]. Nevertheless, in most of these publications the photoactivity of TiO_2_ was determined under UV–vis irradiation [17,26–27,29,31–35]. Much less attention, in contrast, has been paid towards the enhancement of the visible light photoactivity of TiO_2_ by ILs and the elucidation of the role of ILs in the mode of action of photocatalysts [12–13,36–38]. Interestingly, in all mentioned publications [12–13,36–38] the same ionic liquid, 1-butyl-3-methylimidazolium tetrafluoroborate ([BMIM][BF_4_]), was used. However, the proposed mechanisms were different. The photoactivity enhancement under visible-light irradiation was proposed to be due to: (i) doping of nonmetal elements (e.g., N, B, F) derived from the IL structure, inducing a narrower band gap and improving the separation efficiency of the photogenerated electron/hole pairs [13,36–37]; (ii) enhancement of Ti^3+^ species formation in the TiO_2_ lattice (being a source of the electronic charge required for O_2_ adsorption and intermediate energy level) [12,36]; (iii) promoting TiO_2_ hollow structure formation, thereby shortening the diffusion length of the charge carriers as well as increasing the number of reactive sites [12]; and (iv) direct sensitization of TiO_2_ photocatalysts [38]. The only exception is our previous work [14], where three ILs composed of 1-butyl-3-methylimidazolium cation [BMIM] and bromide, hexafluorophosphate [PF_6_] and octylsulphate [OctSO_4_] anions were used. In contrast to [BMIM][BF_4_], the improved TiO_2_ photoactivity under visible-light irradiation originated from the interaction of the bromide anion and molecular oxygen with the TiO_2_ surface with formation of surface complex [14]. Therefore, taking into account the ease of modification of the cation and anion structure of the IL, and as a consequence, the altering of their physicochemical properties, the mechanism of the visible-light activity improvement may vary depending on the IL composition. However, the relation between the IL structure and visible-light photoactivity as well as its mode of action is still not known.

**Figure 1:**
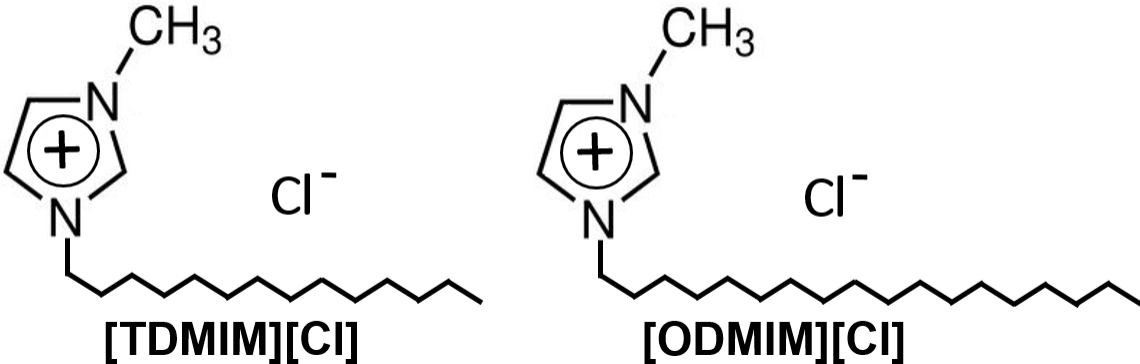
Structures of ionic liquids used in the ionic liquid assisted solvothermal synthesis of TiO_2_–1-methyl-3-tetradecylimidazolium ([TDMIM][Cl]) and TiO_2_–1-methyl-3-octadecylimidazolium chlorides ([ODMIM][Cl]).

In this regard, in this comparative study, we have continued the elucidation of the role of the IL (alkyl chain length in the imidazolium cation) as well as the effect of the IL structure on the formation of TiO_2_ photocatalysts with improved activity towards visible irradiation. The structures of the ILs used in this study, 1-methyl-3-tetradecylimidazolium ([TDMIM][Cl]) and 1-methyl-3-octadecylimidazolium chlorides ([ODMIM][Cl]), are shown in Figure 1. This study gave us better phenomenological insight into the performance of the IL–TiO_2_ photocatalysts and better prospects for optimizing the IL of choice. The results form a part of a very broad, but hardly touched issue in the field of IL–TiO_2_ composites, that is, which structural descriptors of ILs are crucial for the preparation of visible-light-active photocatalysts with desired morphology and properties and how to predict the properties of the IL–TiO_2_ material on the basis of the structure and properties of IL.

## Results and Discussion

Twelve novel TiO_2_ photocatalysts prepared in the presence of two ionic liquids containing different alkyl chain lengths in the imidazolium cation ([ODMIM][Cl] and [TDMIM][Cl]) were obtained by the solvothermal method. All samples were prepared in six different molar ratios IL:TBOT (see Table 1). First of all, the samples were analyzed in terms of specific surface area, optical and photocatalytic properties (in a model reaction of phenol degradation in the aqueous phase under visible-light irradiation). The description of all prepared photocatalysts, including the selected physicochemical and photocatalytic properties, is shown in Table 1. To understand the nature of the visible-light-induced activity (IL:TBOT molar ratio equal to 1:3 and 1:10), the photocatalysts possessing the highest and lowest activity have been selected for further detailed study (SEM, XPS and XRD). Moreover, for the two samples with the highest photoactivity (TiO_2__T(1:3) and TiO_2__O(1:3)), the active species involved in the photocatalytic reaction were also determined.

**Table 1:** Specific surface area (*S*_BET_), pore volume and the efficiency of phenol degradation after 60 min visible-light irradiation of the samples tested in this study.

Sample label	Ionic liquid	Molar ratio (IL:TBOT)	Specific surface area (m^2^·g^−1^)	Pore volume (cm^3^·g^−1^)	Efficiency of phenol degradation (%)

Pristine_TiO_2_	–	–	199	0.10	14
TiO_2__T(1:10)	[TDMIM][Cl]	1:10	211	0.10	23
TiO_2__T(1:8)	[TDMIM][Cl]	1:8	178	0.08	59
TiO_2__T(1:5)	[TDMIM][Cl]	1:5	164	0.08	58
TiO_2__T(1:3)	[TDMIM][Cl]	1:3	156	0.06	61
TiO_2__T(1:2)	[TDMIM][Cl]	1:2	140	0.07	56
TiO_2__T(1:1)	[TDMIM][Cl]	1:1	119	0.05	45
TiO_2__O(1:10)	[ODMIM][Cl]	1:10	193	0.09	23
TiO_2__O(1:8)	[ODMIM][Cl]	1:8	184	0.09	53
TiO_2__O(1:5)	[ODMIM][Cl]	1:5	166	0.08	57
TiO_2__O(1:3)	[ODMIM][Cl]	1:3	157	0.07	59
TiO_2__O(1:2)	[ODMIM][Cl]	1:2	137	0.06	54
TiO_2__O(1:1)	[ODMIM][Cl]	1:1	122	0.05	49

### Morphology and phase structure

**Figure 2:**
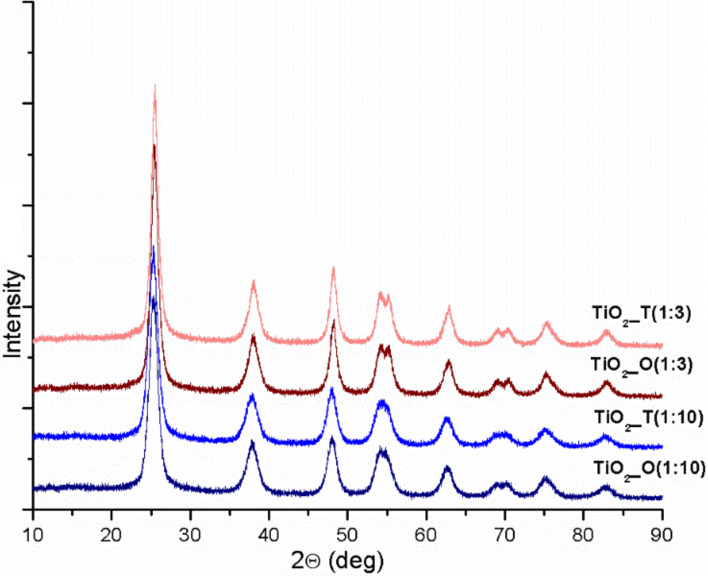
The X-ray diffraction patterns of composite TiO_2_–IL photocatalysts.

The crystal structure of the selected IL–TiO_2_ samples was characterized by XRD, as shown in Figure 2. The typical diffraction peaks corresponding to anatase phase of TiO_2_ (2θ = 25.3°, 37.8°, 48.1°, 54°, 54.9°, 62.7°, 68.5°, 70.2°, 75°, 82.6°) were observed for all photocatalysts. The analysis confirmed that the samples do not contain any impurities and anatase phase of high quality was formed. Based on the line width analysis of the anatase (101) reflection peak, the average crystal size of the crystallites (*d*) forming photocatalysts with the highest (IL:TBOT molar ratio 1:3) and the lowest photoactivity (IL:TBOT molar ratio 1:10), estimated by the Scherrer equation, were determined and summarized in Table 2.

**Table 2:** Lattice parameters and average crystallite size of the IL–TiO_2_ photocatalysts. The values in parenthesis represent the error in measurement.

Sample label	*a* (Å)	*c* (Å)	*V* (Å^3^)	*d* (Å)

TiO_2_	3.7890(3)	9.497(1)	136.34(4)	63
TiO_2__O(1:10)	3.7816(1)	9.520(8)	136.15(3)	57
TiO_2__O(1:3)	3.7692(9)	9.522(8)	135.29(6)	78
TiO_2__T(1:10)	3.7760(8)	9.530(3)	135.89(1)	74
TiO_2__T(1:3)	3.7687(7)	9.518(2)	135.19(3)	81

The average crystal size values of the IL–TiO_2_ composites generally increased with the addition of ILs in comparison to pristine TiO_2_. This could suggest that the addition of ILs during the preparation of TiO_2_ affects the growth of the anatase nanocrystals, forming microparticles. Similar results were reported by Li et al. [37] who suggest that [BMIM][BF_4_] acted as a growth inhibitor of the anatase crystal. The addition of ILs to the TiO_2_ preparation route also influenced the length of the cell edges – a decrease of *a* and increase of *c* lengths in comparison to pristine TiO_2_ was detected. These observations can be related to changes in the crystalline structure, i.e., generation of Ti^3+^ as will be described below in the next section. Comparing the IL–TiO_2_ samples, the photocatalysts with the highest activity were characterized by the shorter length of both cell edges as well as the lowest cell volume (Table 2).

**Figure 3:**
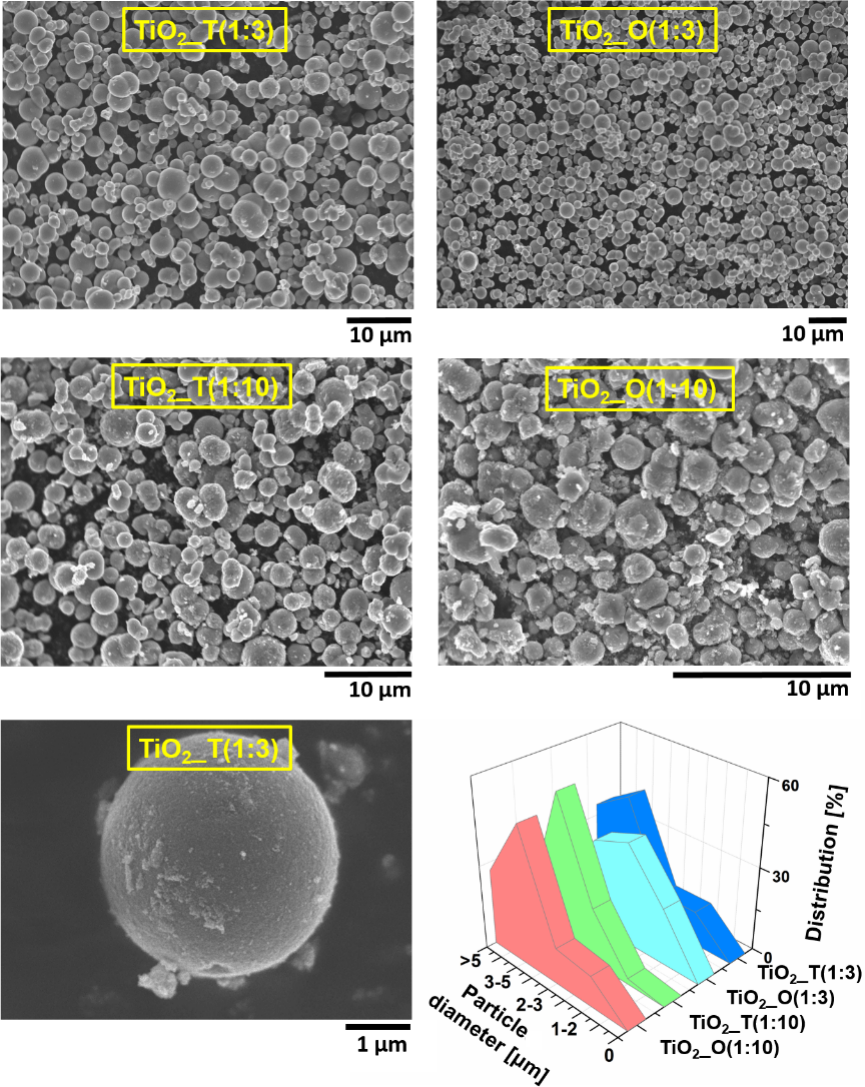
SEM images and particles size distribution of ILs assisted TiO_2_microspheres.

The surface morphology and microstructure of the IL–TiO_2_ composites were characterized by SEM. The morphology of four selected samples prepared with different IL concentrations (IL:TBOT molar ratio equal to 1:3 and 1:10) which revealed the highest and the lowest photoactivity under visible-light irradiation is presented in Figure 3. The average size of the microspheres, calculated based on the statistical average size of 100 microstructures, is also included in this figure. The four main fractions of TiO_2_ microspheres have been recognized. Generally, for all the samples, the dominant fraction were the microspheres with an average diameter ranging from 3 to 5 µm. Based on these results, as well as our previous study [28], we can conclude that longer alkyl chains in the imidazolium cation lead to a larger particle size. Recently we examined the influence of two ionic liquids with four and ten atoms of carbon in the alkyl chain of the imidazolium cation (i.e., 1-butyl- and 1-decyl-3-methylimizadolium chlorides, [BMIM][Cl] and [DMIM][Cl], respectively) on the selected properties and photoactivity of TiO_2_ particles under UV irradiation. For [BMIM][Cl] (IL:TBOT molar ratio 1:10), the highest contribution (44%) had structures with diameter 0.5–1 μm, whereas for [DMIM][Cl], up to 50% were particles of 1–2 μm. For comparison, IL–TiO_2_ structures prepared in this study in the presence of [TDMIM][Cl] and [ODMIM][Cl], for a IL:TBOT molar ratio equal to 1:10, yielded mainly particles with a diameter of 3–5 μm, in an amount of 54% and 49%, respectively. It was also observed that synthesis in the presence of low IL content in the reaction mixture (i.e., IL:TBOT molar ratio of 1:10) provided TiO_2_ structures with deformations (Figure 3), whereas the photocatalysts prepared using a IL:TBOT molar ratio equal to 1:3 (TiO_2__T(1:3) and TiO_2__O(1:3)) had a more uniform, spherical shape and smoother surface. Moreover, increasing the concentration of IL resulted in formation of particles with a higher contribution of smaller particles in comparison to samples obtained using IL:TBOT with a molar ratio equal to 1:10. [TDMIM][Cl] and [ODMIM][Cl] are salts consisting of a large, asymmetric, amphiphilic imidazolium cation and a small single-atomic chloride anion. Based on our previous research we can assume that ILs interact with the TiO_2_ surface, making a protective layer due to a combination of electrostatic and steric stabilization. 1-Methyl-3-octadecylimidazolium chloride is probably less firmly packed on the TiO_2_ surface due to the longer alkyl chain. As a result, the [ODMIM][Cl] ionic liquid may limit smaller particle deposition during Ostwald ripening, thus inhibiting further growth and agglomeration. For the TiO_2__O(1:3) sample, the dominant fraction were microspheres with diameter ranging from 2–3 μm (38%), whereas for TiO_2__T(1:3), the main fraction was particles with an average diameter of 3–5 μm (42%).

### Diffuse reflectance spectroscopy, UV–vis spectroscopy and BET analysis

The Brunauer–Emmett–Teller (BET) specific surface area (*S*_BET_) of the IL–TiO_2_ photocatalysts ranged from 119 to 211 m^2^·g^−1^ (see Table 1). For comparison, pristine TiO_2_ obtained by the same method but without IL, has a *S*_BET_ equal to 199 m^2^·g^−1^. In this regard, all samples (except TiO2_T(1:10) ) had a smaller *S*_BET_ as compared to TiO_2_ unmodified by ILs. Taking into account the type and the amount of IL used in the experiments, the largest *S*_BET_ was detected for the IL–TiO_2_ samples obtained with the IL:TBOT molar ratio of 1:10, that is, the lowest amount of IL used for TiO_2_ synthesis. The specific surface area determined for these samples was 211 m^2^·g^−1^ and 193 m^2^·g^−1^ for [TDMIM][Cl] and [ODMIM][Cl], respectively. Further increase of the IL content in the reaction mixture resulted in decrease of *S*_BET_. The results obtained for both IL are similar (Table 1), therefore influence of the chain length in the imidazolium cation on the surface properties of IL–TiO_2_ was not observed. However, the comparison of *S*_BET_ of samples described in this work with values previously presented by Paszkiewicz [28] for ILs containing shorter chain lengths in the imidazolium cation (C4 and C10) revealed that elongation of the alkyl substituent provided photocatalysts with smaller surface area. This property may be related to formation of the protecting layer by the IL at the TiO_2_ surface, resulting therefore in higher steric hindrance for salts with longer alkyl chains influencing the final pore volume, and thus, the surface area. The total pore volume ranged from 0.05 to 0.1 cm^3^·g^−1^ depending on the IL type used in the experiments and its concentration. A higher BET surface area was also related to higher pore volume values and could lead to improved efficiency of the photocatalytic reactions [39].

**Figure 4:**
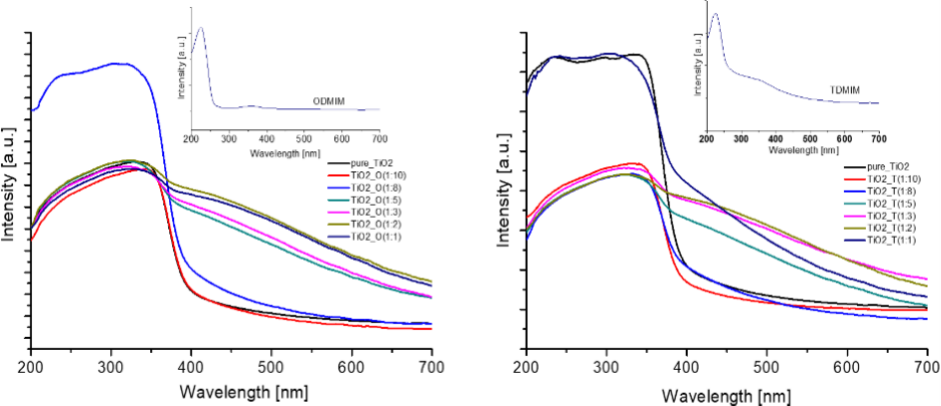
The diffuse reflectance and UV–vis spectra for the samples ODMIM_Cl_TiO_2_ (left) and TDMIM_Cl_TiO_2_ (right).

The diffuse reflection (DR)/UV–vis spectra of the photocatalysts as well as for the pure ionic liquids used in this study are shown in Figure 4. The pristine TiO_2_ obtained by the solvothermal method showed the expected bandgap of 3.2 eV and very weak visible-light absorption. However, the visible-light absorption of the TiO_2_ photocatalysts appeared when the selected ionic liquids, [ODMIM][Cl] and [TDMIM][Cl], were applied in the TiO_2_ synthesis reaction. Moreover, with increasing IL:TBOT molar ratio, the optical absorption of the composites in the viible-light region was also greatly increased (compare for example spectra for IL–TiO_2_ obtained at IL:TBOT molar ratio of 1:10 and 1:3). Therefore, this enhanced absorbance of the light in the visible spectrum is expected to enhance the photocatalytic activity in the visible region for the target reaction. This observation is well verified by the photocatalytic tests of the IL–TiO_2_ composites for the phenol degradation under visible light irradiation (Table 1).

Additionally, to understand why the obtained microparticles exhibited a strong light absorption over the visible range pure ILs were also examined as shown in Figure 4. The shape and nature of the imidazolium ionic liquid spectra are consistent with literature data [40–41]. A clear maximum is observable above 220 nm and a long tail of the absorption extended even beyond 350 nm and 500 nm for [ODMIM][Cl] and [TDMIM][Cl], respectively. The intense absorption in the UV region can be assigned to the π–π* transition of the imidazolium ring. On the other hand, the long tail of the absorption spectra could be due to (i) the energy structure of the cation as well as the spatial arrangement of the anion and (ii) the presence of an impurity resulting from the IL synthesis [42–44].

Considering the above results the improvement of the optical absorption of the composites in the visible-light region could be associated with the synergic effect of its two main components, that is, TiO_2_ and ILs. Binetti et al. [42] stated that the absorbance of IL–TiO_2_ materials increased with the modification of TiO_2_ with 1-hexyl-3-methylimidazolium tetrafluoroborate ([HMIM][BF_4_]), and concomitantly, a tail appears in the visible region due to absorption by IL. Moreover, taking into account that both ILs used in this study have a high number of carbon atoms in the structure (Figure 1), thus high carbon content, the absorption of IL–TiO_2_ in the visible region can be attributed to the existence of carbon species on the surface of the photocatalysts [45].

### Chemical composition of ionic liquid/TiO_2_ composites

**Figure 5:**
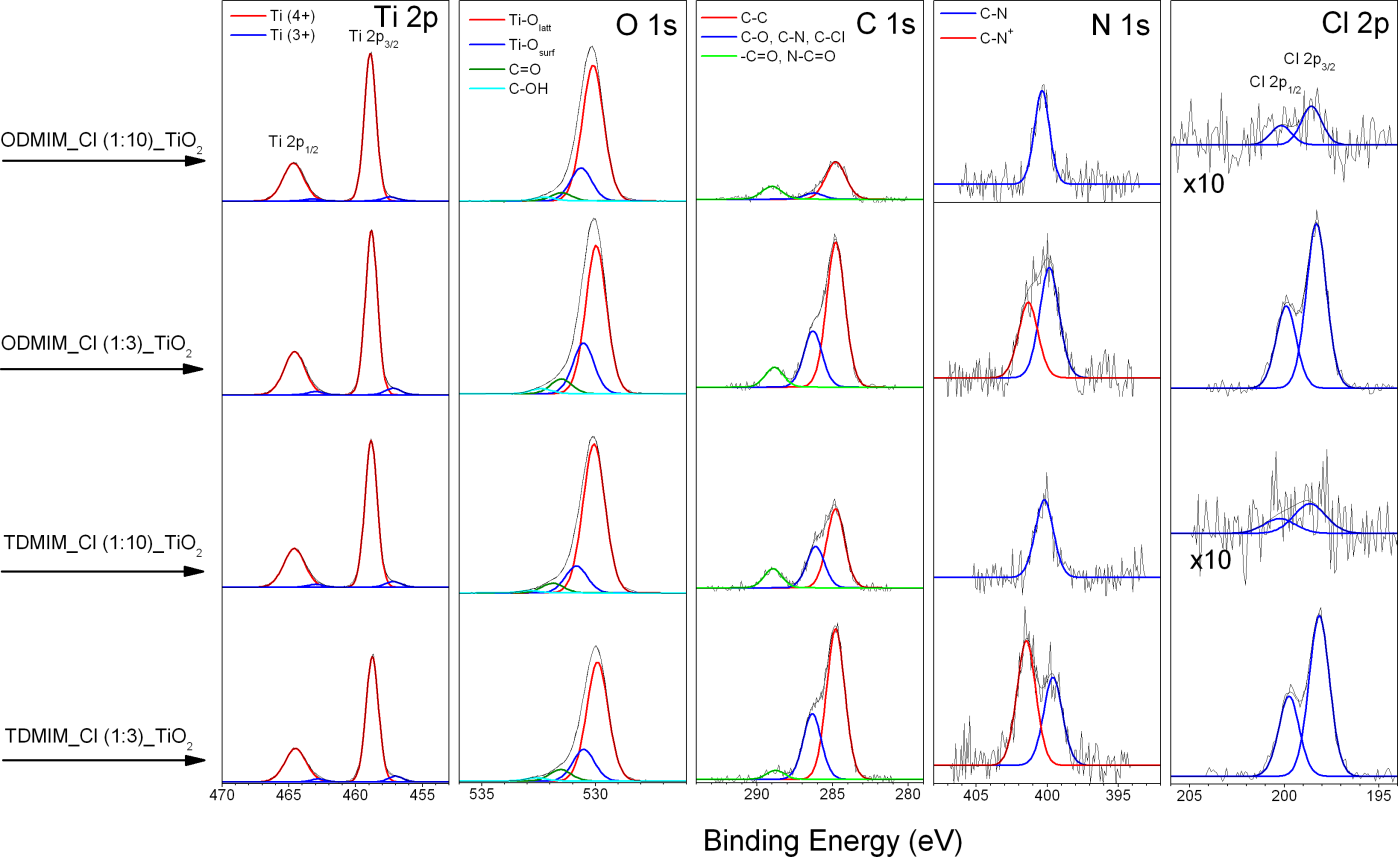
High-resolution XPS spectra of elements detected in the [ODMIM][Cl]–TiO_2_ and [TDMIM][Cl]–TiO_2_ samples.

The photocatalytic degradation is based on chemical reactions on the surface of the photocatalyst [46–48]. In this context, XPS measurements have been chosen to analyze the surface of the IL–TiO_2_ composites. Both [ODMIM][Cl]–TiO_2_ and [TDMIM][Cl]–TiO_2_ samples modified with a IL:TBOT molar ratio of 1:3 and 1:10 were investigated. The elemental surface composition of all IL–TiO_2_ specimens analyzed is shown in Table 3. The titanium, oxygen, carbon, nitrogen and chlorine atoms were detected. The last two elements evidenced successful interaction between the IL and the surface of TiO_2_. The chemical character of these elements was identified from corresponding Ti 2p, O 1s, C 1s, N 1s and Cl 2p high-resolution XPS spectra, respectively. The deconvoluted spectra are shown in Figure 5. The carbon fraction at binding energy (BE) close to 286.2 eV is characteristic for C–Cl and C–N bonds [49]. Two nitrogen states can be distinguished in the N 1s spectra recorded on the IL–TiO_2_ composites (see Figure 5 and Table 3). The signal at BE = 400 eV can be assigned to nitrogen states formed by Ti–O–N and C–N bonds [50]. The last one can be from superposition of C–NH–C and C=N–C bonds attributed to pyrrole- and pyridine-type interactions [49,51–52]. The peaks at BEs abov**e** 401.4 eV can be due to the positively charged nitrogen (N^+^) caused by pyridine-type nitrogen bonds and oxidized nitrogen species [53]. It should be noted that this nitrogen state appears for both types of IL–TiO_2_ with a molar ratio of IL:TBOT of 1:3. No signals located at BEs lower than 399 eV, characteristic for Ti–N bond formation [28], were detected. The XPS data collected in Table 3 showed that the amount of carbon, nitrogen and chlorine were higher for the samples with molar ratio 1:3 than for those with molar ratio 1:10. In addition, the surface concentration of these elements for the TiO_2__O(1:3) sample with longer alkyl substituents is smaller than corresponding values evaluated for TiO_2__T(1:3) samples containing ILs with shorter substituents (see the C/Ti and N/Ti entries in Table 3). Thus, the lower coverage of the [ODMIM] cations seems to be the result of the larger steric effect induced by this ion. The C/N ratios, evaluated for all samples, were estimated to be higher than nominal values related to the respective IL cations (see Table 3). This may be caused by adsorption of additional carbon contaminants on the finally prepared IL-assisted TiO_2_ because of the exposure of the samples to air prior to the XPS analysis or during synthesis and preparation.

**Table 3:** Elemental composition (in atom %) and chemical characteristics of titanium, oxygen, carbon and nitrogen states in the surface layer of [ODMM][Cl] and [TDMM][Cl] IL-modified TiO_2_ particles, evaluated by X-ray photoelectron analysis.

Sample	TiO_2__O(1:10)	TiO_2__O(1:3)	TiO_2__T(1:10)	TiO_2__T(1:3)

∑Ti	26.97	24.38	25.66	23.79
Ti^(4+)^ 458.9 ± 0.2 eV	96.18	94.95	94.87	94.55
Ti^(3+)^ 457.2 ± 0.2 eV	3.82	5.05	5.13	5.45

∑O	69.22	64.05	67.05	61.79
Ti–O_latt_ 530.0 ± 0.1 eV	74.98	67.79	79.14	71.77
Ti–O_surf_ 530.6 ± 0.2 eV	18.36	23.18	14.35	19.17
C=O 531.6 ± 0.2 eV	4.53	6.72	5.26	7.06
–OH 532.6 ± 0.2 eV	2.12	2.31	1.25	2

∑C	3.51	9.84	7	12.22
C–C 284.8 ± 0.1 eV	66.09	65.54	56.57	66.8
C–OH, C–Cl, C–N 286.2 ± 0.1 eV	10.9	25.49	29.71	29.22
–C=O, N–C=O 289.0 ± 0.1 eV	23	8.96	13.72	3.99

∑N	0.26	0.52	0.24	0.72
“A” 400.0 ± 0.4 eV	100	59.42	100	41.39
“B” 401.4 ± 0.1 eV	0	40.58	0	58.61

∑Cl	0.05	1.21	0.06	1.48
C/N	13.5	18.9	29.2	17
N/Ti	0.01	0.021	0.009	0.03
C/Ti	0.13	0.4	0.27	0.51
Cl/N	0.19	2.33	0.25	2.06

### Photocatalytic activity

**Figure 6:**
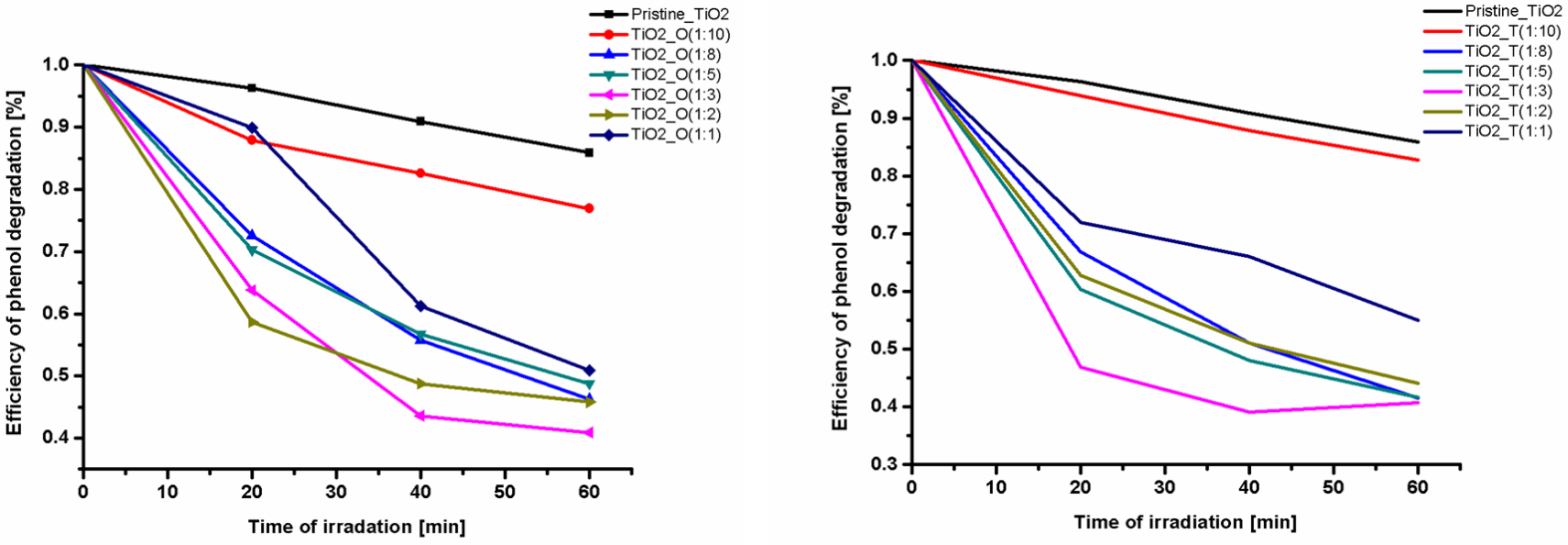
The efficiency of phenol degradation for ODMIM_Cl_TiO_2_ (left) and TDMIM_Cl_TiO_2_ (right) photocatalyst samples.

The photocatalytic activity of the IL–TiO_2_ samples was evaluated by phenol degradation induced by visible-light irradiation (using an optical filter with λ > 420 nm). All photocatalysts exhibited better photocatalytic properties than the reference TiO_2_ sample (Table 1 and Figure 6).

Before the photocatalytic activity tests, phenol adsorption on the surface of the photocatalyst was tested for the samples possessing the highest photoactivity. It was observed that the concentration of phenol after 120 min in dark conditions practically did not change. The samples with the highest photoactivity were prepared using a IL:TBOT molar ratio of 1:3. The efficiency of phenol degradation increased from 14 to 59% and 61% for pristine TiO_2_, TiO_2__O(1:3) and TiO_2__T(1:3) samples, respectively. Moreover, for both ionic liquids the phenol degradation efficiency increased with increasing IL:TBOT molar ratio from 1:10 to 1:3. However, a further increase in the IL concentration in the reaction system resulted in an opposite relation. These results corresponded well with SEM and XRD results. Particles prepared using a IL:TBOT molar ratio equal to 1:10 were poorly formed and had highly irregular shapes, which resulted in lower photocatalytic activity. Based on the literature data, it is known that regularly shaped TiO_2_ microspheres show a higher photoefficiency due to their low density, high surface-to-volume ratio, high surface area and good surface permeability [54–56]. Thus, it could be expected that the higher photocatalytic activity could be achieved by using TiO_2_ microspheres with uniform and spherical shape.

According to the DRS UV–vis spectra shown in Figure 4, the incorporation of ILs into TiO_2_ microspheres significantly extended the absorption spectrum of the titanium dioxide in the visible region. Therefore, the enhanced photocatalytic activity of IL–TiO_2_ can be attributed to the improved optical absorption. Moreover, the XPS data showed that the amount of carbon, nitrogen and chlorine were higher for the samples with molar ratio 1:3 in comparison to 1:10, which corresponds well with changes in photoactivity. It should be noticed that the total amount of C, N and Cl was higher for TiO_2__T(1:3) than TiO_2__O(1:3) despite that the [ODMIM] cation contained a longer alkyl chain than [TDMIM]. This is probably due to the higher steric hindrance created by [ODMIM][Cl] at the TiO_2_ surface, hence a probably less firmly packed organic protection layer. Additionally, the XPS analysis also confirmed that C, N and Cl atoms are located solely on the surface of the semiconductors. Hereby, the possible mechanism of the TiO_2_ photoactivity improvement under visible-light irradiation by doping of nonmetal elements derived from IL structures was rejected.

Therefore, to further elucidate the mechanisms of the photocatalytic reaction, the role of the generated reactive species in the photocatalytic process under visible irradiation was investigate using *t*-BuOH, AgNO_3_, *p*-benzoquinone (BQ) and ammonium oxalate (AO) as the scavengers of ^•^OH, e^−^, O_2_^−•^ and h^+^, respectively. These measurements have been performed for the IL–TiO_2_ samples possessing the highest activity, that is, TiO_2__T(1:3) and TiO_2__O(1:3), and the obtained results are presented in Table 4. It was observed that application of AgNO_3_ as a scavenger of e^−^ for the both samples (TiO_2__T(1:3) and TiO_2__O(1:3) did not affect the effectiveness of the photocatalytic process compared to that carried out without scavengers.

**Table 4:** Percent efficiency of phenol degradation under visible light in the presence of scavengers.

Sample ID	AgNO_3_	C_2_H_8_N_2_O_4_	C_4_H_9_OH	C_6_H_4_O_2_

TiO_2__T(1:3)	68	54	63	22
TiO_2__O(1:3)	77	53	85	11

Participation of O_2_^•^ in the photodegradation process was investigated by adding BQ, which is capable of trapping O_2_^•^ [57]. It was observed that the addition of BQ to both samples (TiO_2__O(1:3) and TiO_2__O(1:3)) caused inhibition of phenol degradation, as presented in Table 4. The degradation rate was largely suppressed to 22 and 11% for the TiO_2__T(1:3) and TiO_2__O(1:3) photocatalysts, respectively. It is deduced that the oxidation inhibition of phenol is due to the suppression of the superoxide anion formation by the BQ addition. Addition of AO (holes scavenger) resulted in a slight inhibition of the photodegradation process under visible-light irradiation for both samples, indicating that h^+^ might play a negligible role in the phenol photo-oxidation process. Probably the positively charged hole could catch OH^−^ to yield hydroxyl radicals [58]. In the case of the TiO_2__T(1:3) sample, when *tert*-butyl alcohol (^•^OH scavenger) was added to the phenol solution, 63% of phenol was degraded, indicating that OH radicals play an insignificant role in the degradation process. Interestingly, for the TiO_2__O(1:3) photocatalyst, the addition of AgNO_3_ and *tert*-butyl alcohol increased the effectiveness of the photocatalytic process. As explained by Liu et al., AgNO_3_ may be capable of enhancing the separation of electrons and holes, resulting in a greater amount of holes and following active species generated, which promotes the degradation rate [59].

## Conclusion

The results presented in this study revealed that visible-light-responsive TiO_2_ microspheres could be successfully obtained by a solvothermal method assisted by ionic liquids, such as 1-methyl-3-tetradecylimidazolium or 1-methyl-3-octadecylimidazolium chlorides. The ionic liquids introduced into the reaction environment work as structure-controlling agents, providing TiO_2_ microparticles (composed of nanocrystals) with a large specific surface area ranging from 119 to 211 m^2^/g. However, more importantly, application of ionic liquids during TiO_2_ synthesis enabled formation of visible-light-induced photocatalysts. It was found that the amount of the ionic liquid used for the synthesis is crucial and significantly affects the photoresponse of the IL–TiO_2_ materials toward visible irradiation, thus photoactivity. The highest photoactivity was observed for samples prepared using a IL:TBOT molar ratio of 1:3, as observed for both ILs. From 59% to 61% of phenol used as a model compound was degraded after 60 min of irradiation by visible light (λ > 420 nm) in the presence of suspended TiO_2_ that was modified by [ODMIM][Cl] and [TDMIM][Cl], respectively. The enhanced photocatalytic activity of IL–TiO_2_ can be attributed to the presence of the ionic liquid cation and anion at the TiO_2_ surface, resulting in the improved optical absorption of visible light by the photocatalysts. The phenol degradation was realized mainly by radical anions O_2_^•−^, whereas the contribution of the other active species was limited in the reaction mechanism. In this regard, the role of the ionic liquid in TiO_2_ photoexcitation requires further investigation.

## Experimental

### Chemicals

Titanium(IV) *n*-butoxide (TBOT), (≥97%) as a precursor of TiO_2_, hydrochloric acid (36%) as a pH stabilizer and ethanol (99.9%) as the reaction medium were provided by Sigma-Aldrich. The ionic liquids, 1-methyl-3-octadecylimidazolium chloride [ODMIM][Cl] and 1-methyl-3-tetradecylimidazolium chloride [TDMIM][Cl] were purchased from Ionic Liquids Technologies GmbH. Ammonium oxalate, silver nitrate (≥99%), benzoquinone and *tert*-butyl alcohol from Sigma-Aldrich were used as scavengers.

### Photocatalyst preparation

TiO_2_ was modified by ILs using a solvothermal method. First of all, the titania precursor, Ti(IV) *n*-butoxide, was dissolved in absolute ethanol under stirring. Then the hydrochloric acid, distilled water and selected ionic liquid were added. Vigorous stirring of the solution was continued for 10 min to obtain a transparent solution. In the final step, the homogeneous mixture was transferred to a 200 mL teflon-lined stainless steel autoclave and the solvothermal reaction was performed at 180 °C for 24 h [28]. The reaction product was washed with ethanol and deionized water, respectively, followed by drying at 60 °C for 6 h. The obtained powder was calcinated at 200 °C for 2 h (heating rate 2 °C/min). Various molar ratios of ILs to TBOT were selected as listed in Table 1.

### Sample characterization

The morphology and size distribution of the TiO_2_ powders were observed using a Jeol SEM microscope operated at 12 kV and Cs-corrected STEM (high angle annular darkfield, HAADF).

X-ray photoelectron spectroscopy (XPS) experiments were performed on a PHI 5000 VersaProbeTM (ULVAC-PHI) spectrometer with monochromatic Al Kα radiation (*h*ν = 1486.6 eV). The high-resolution (HR) XPS spectra were collected by the hemispherical analyzer at a pass energy of 23.5 eV and an energy step size of 0.1 eV. CasaXPS software was used to evaluate the XPS data. The binding energy (BE) scale of all detected spectra was referenced by setting the BE of the aliphatic carbon peak (C–C) signal to 284.8 eV.

The UV–vis reflectance and absorbance spectra of TiO_2_ photocatalysts were recorded using a Shimadzu UV–vis spectrophotometer (UV 2600) equipped with an integrating sphere and BaSO_4_ was used as the reference sample.

BET surface area and total pore volume of the photocatalysts (physical adsorption and desorption of nitrogen at 77 K) was measured using a Micromeritics Gemini V200 instrument. The photocatalyst samples were dried and degassed in a sample cell at 200 °C for at least 2 h before the adsorption. The specific surface area of the photocatalysts was determined by the BET method.

Powder X-ray diffraction (XRD) studies of the photocatalyst samples were carried out using a RigakuMiniFlex 600 XRD system equipped an X-ray generator with a copper target (operated at 40 kV and 30 mA). The average TiO_2_ crystallite size was calculated using the Scherrer equation.

### Photocatalytic activity test

For determination of the photoactivity under visible-light irradiation, the aqueous phase containing 125 mg of the photocatalyst (5 g/dm^3^), 24 cm^3^ of deionized water and 1 cm^3^ of phenol as a model compound (Co = 500 mg/dm^3^) was used. The suspension was stirred and aerated (*V* = 5 dm^3^/h) for 30 min in the dark to obtain equilibrium and then the content of the reactor was photoirradiated with a 1000 W Xe lamp (Oriel, light flux 6.5–7.5 mW/cm^2^) which emitted both UV and visible-light irradiation. The photoreactor (*V* = 25 cm^3^, i.d. 37 mm, length 30 mm) was equipped with a quartz window and exposure layer thickness was 3 cm. The optical path included a water filter and a glass filter (GG 420) which cut-off wavelengths shorter than 420 nm. The temperature of the aqueous phase during irradiation was kept at 10 °C using a water bath. During the irradiation, the suspension (1 cm^3^) was collected and filtered through syringe filters (diameter 0.2 µm) to remove the photocatalyst particles. The phenol concentration was estimated by means of the colorimetric method (λ = 480 nm) after derivatization with diazo-*p*-nitroaniline using a UV–vis spectrophotometer (Thermo Evolution 220). The photocatalytic degradation runs were preceded by a blind test in the absence of a photocatalyst or illumination. No degradation of phenol was observed in the absence of either the photocatalyst or illumination.

### Determination of reactive species

To clarify which active species are involved in the photodegradation of the model compound, the phenol irradiation process was preceded by analogous experiments performed in the presence of different radicals scavengers (ammonium oxalate as a scavenger for photogenerated holes, AgNO_3_ as scavenger for electrons, benzoquinone (BQ) as scavenger for superoxide radical species, and *tert*-butyl alcohol as a scavenger for hydroxyl radical species). The procedure of the photocatalytic measurements was carried out similar to the above-described photocatalytic degradation of phenol, except that the radical scavengers were added to the reaction system. The scavenger concentration was equal to 0.21 mmol dm^−3^. No adsorption of phenol was observed in the presence of the photocatalyst in the phenol/scavenger solution and absence of illumination.
